# Correlation between Tumor Microenvironment and Immune Subtypes Based on CD8 T Cells Enhancing Personalized Therapy of Gastric Cancer

**DOI:** 10.1155/2022/8933167

**Published:** 2022-02-28

**Authors:** Jianyu Wu, Yajie Xiao, Weiqi Lu, Zijing Zhang, Haigan Yang, Xiaoli Cui, Dongfang Wu, Yuzhong Chen

**Affiliations:** ^1^No. 2 Surgery Department, The First Affiliated Hospital of Guangzhou University of Traditional Chinese Medicine, Guangzhou 440100, China; ^2^YuceBio Technology Co., Ltd., Shenzhen 440300, China

## Abstract

**Background:**

Immunotherapy is a promising therapy for metastatic gastric cancer (GC) patients. However, the component of tumor microenvironment (TME) is a pivotal factor hindering immunotherapy outcome. CD8 T cells suppress tumor progression. This study developed an immune subtyping system and a prognostic model for guiding personalized therapy of GC patients.

**Methods:**

Marker genes related to CD8 T cells were identified by weighted correlation network analysis (WGCNA). Consensus clustering was used to develop immune subtypes. Univariate Cox regression analysis was performed to screen prognostic genes. Functional analysis (KEGG and GO annotation) and gene set enrichment analysis were applied.

**Results:**

Based on marker genes related to CD8 T cells, we identified three immune subtypes (IC1, IC2, and IC3) with distinct prognosis and differential TME. In IC3, CD8 T cell function was impaired by high activation of CXCR4/CXCL12 axis, and impaired T cell function predicted high response to immune checkpoint blockade. IC1 was sensitive to chemotherapeutic drugs but showed low response to immunotherapy. We also developed an 8-gene prognostic signature with robust performance to stratify GC patients into high-risk and low-risk groups.

**Conclusions:**

This study identified three immune subtypes and a prognostic signature, and both were effective in direct personalized therapy for GC patients. The correlation between TME and immunotherapy was further characterized from a new perspective.

## 1. Introduction

Although the incidence and mortality of gastric cancer (GC) have declined over the past decades, GC is still the leading cause of cancer death [1]. The discovery and application of curative modalities for GC treatment increased the 5-year overall survival (OS) rate from 18.8% to 28.0% according to the statistics of the Surveillance, Epidemiology, and End Results (SEER) program [2]. However, a large number of metastatic patients still face the difficulties of seeking an effective therapy. Currently, immunotherapy targeting immune checkpoints seems a promising strategy for treating advanced gastric cancer [3].

Tumor microenvironment (TME) is highly associated with tumor cell proliferation, invasion, migration, and immunotherapy outcome [4, 5]. To a large extent, infiltration of different types of immune cells is decisive of the prognosis of immunotherapy [6]. An extensive immunogenomic analysis on pan-cancer performed with The Cancer Genome Atlas (TCGA) identified 6 immune subtypes, and GC can be classified into 5 immune subtypes [7]. The pan-caner study further characterized the critical role of TME to drive personalized cancer immunotherapy. Focusing on tumor immune infiltration in gastric cancer, Zhou et al. developed two immune subtypes (Immune Activation Subtype and Immunosuppressive Subtype), which were predicted to have different responses to different immunotherapies [8].

A link between increased levels of cytotoxic CD8 T cells and strong antitumor effects has been discovered in many cancer types such as breast cancer [9], glioblastoma, cervical cancer [10, 11], and gastric cancer [12]. In the TME, receptors of PD-L1 and CD80 expressed by tumor cells or tumor-related immune cells can interact with PD-1 and CTLA-4 expressed by CD8 T cells, respectively, to impair CD8 T cell function [13, 14]. These interactions may be the potential targets for immunotherapy [15, 16]. Current studies also proved that anti-PD-1/PD-L1 and anti-CTLA-4 inhibitors can suppress cancer cell proliferation [17]. Clinical trial of anti-PD-1 antibody combined with apatinib revealed a positive outcome in advanced GC patients [18]. Immune infiltration of CD8 T cells plays a pivotal role in inhibiting cancer cell progression, and its function is closely correlated with TME. In addition, immune response activated by targeted immunotherapy is highly related to the status of infiltrated CD8 T cells and TME [19].

The current study focused on CD8 T cells and examined the role of CD8 T cells in immunotherapy. Integrative bioinformatics analysis identified genes related to CD8 T cells, based on which three immune subtypes with distinct prognosis were determined. A link between immune subtypes and personalized therapy such as immunotherapy was comprehensively described in the study. Furthermore, we constructed an 8-gene prognostic signature to predict the outcomes of GC patients and guide immunotherapy.

## 2. Materials and Methods

### 2.1. Data Information and Study Design

**Figure 1:**
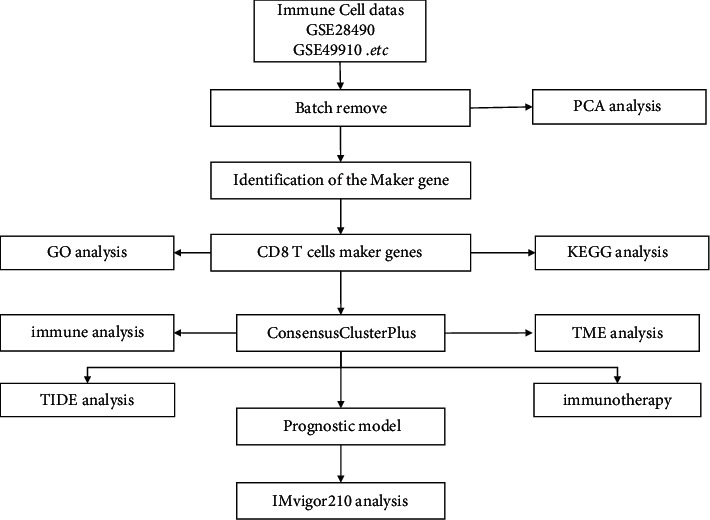
The flow chart of developing immune subtypes and prognostic genes based on genes related to CD8 T cells.

GC samples and expression data of immune cells were obtained from public databases. TCGA-STAD dataset was downloaded from TCGA (https://portal.gdc.cancer.gov/). GSE26942 [20], GSE66229 [20], and GSE84437 [21] datasets containing GC samples were downloaded from Gene Expression Omnibus (GEO, https://www.ncbi.nlm.nih.gov/geo/). Expression data of immune cells was obtained from GEO, including GSE13906 [22], GSE23371 [23], GSE27291 [24], GSE27838 [25], GSE28490 [26], GSE28726 [27], GSE37750 [28], GSE39889 [29], GSE42058 [30], GSE49910 [31], GSE59237 [32], GSE6863 [33], and GSE8059 [34] (Supplementary Table S1). GSE78220 [35] contains the immunotherapy data of metastatic melanoma patients. IMvigor210 [36] dataset was from https://research-pub.gene.com/IMvigor210CoreBiologies. The workflow of this study is shown in Figure 1.

### 2.2. Data Preprocessing

Of TCGA-STAD dataset, samples without survival status, survival time, or follow-up data were excluded. Using R software package hgu133plus2.db to convert Ensembl ID to gene symbol, genes with relative expression level <1 in over 50% samples were excluded. The median of expression was selected when one gene had more than one gene symbol. In this way, 353 samples were included in TCGA-STAD dataset (Supplementary Table S2). Of GC samples in GSE cohort, normal samples, and samples without survival status, survival time or follow-up data were excluded. Genes in probes were converted to gene symbol. Finally, 826 samples were included in the GSE cohort (Supplementary Table S2).

The RMA procedure in affy package [37] was used process raw data of Affymetrix GeneChip data for GSE cohort. Then, batch effect among different batches was removed using the function “removeBatchEffect” in limma R package [38]. The principle component analysis (PCA) was applied to display the expression data before and after the removal of batch effect. No difference was observed in TCGA-STAD datasets and immune cell datasets after removing the batch effect (Supplementary Figures S1 and S2).

### 2.3. Weighted Correlation Network Analysis (WGCNA)

WGCNA was applied to identify coexpression gene modules from immune cell data, and to construct weight coexpression networks [39]. Pearson correlation coefficients between genes were calculated. The optimal power of soft threshold (*β*) was confirmed, according to the coefficient between log (*k*) and log (*p*(*k*)). For a scale-free network, the coefficient between log (*k*) and log (*p*(*k*)) up to 0.85 was selected. Then, expression matrix was converted to adjacent matrix and topological overlap matrix (TOM). Using average-linkage hierarchical clustering, genes were clustered with at least 100 genes in one gene module based on hybrid dynamic shear tree and TOM. Gene modules were further clustered according to the eigengenes of each module under the criteria of height = 0.25, deepSplit = 2, minModuleSize = 150.

### 2.4. Gene Enrichment Analysis

R package of clusterProfiler (v3.14.0) was employed to annotate Kyoto Encyclopedia of Genes and Genomes (KEGG) pathways and gene ontology (GO) terms of marker genes related to CD8 T cells [40]. CIBERSORT [41] (https://cibersort.stanford.edu/) was used to calculate the enrichment score of 22 types of immune cells. GSVA R package was applied for single sample gene set enrichment analysis (ssGSEA) to analyze the relation between risk score and KEGG pathways [42].

### 2.5. Identification of Immune Subtypes Based on CD8 T Cells

Marker genes related to CD8 T cells were identified by WGCNA. Univariate Cox regression analysis screened genes related to prognosis from TCGA-STAD dataset and GSE cohort. The intersected genes between the two datasets were selected for consensus clustering in TCGA-STAD dataset. ConsensusClusterPlus R package was applied to perform unsupervised consensus clustering [43]. The optimal cluster number *k* = 3 was confirmed by cumulative distribution function (CDF) and the relative change in area under CDF curve. Kaplan-Meier survival analysis was performed to verify the effectiveness of classification. GSE cohort was used to validate the robustness of classification.

### 2.6. The Relation between Immune Subtypes and Personalized Therapy

TIDE (https://tide.dfci.harvard.edu/) was used to predict the potential correlation between immune subtypes and immune response. Higher TIDE score integrating T cell dysfunction and T cell exclusion was positively related to the possibility of immune escape. GSE78220 dataset containing anti-PD-1 immunotherapy data of melanoma patients was used for submap analysis on TCGA-STAD samples. Lower *p* value represented a higher similarity of treatment outcomes among samples. Bonferroni-correction was performed to correct *p* value. In addition, estimated IC50 of chemotherapeutic drugs including cisplatin, cyclopamine, and rapamycin was analyzed in different immune subtypes. Lower estimated IC50 represented higher drug sensitivity.

### 2.7. Construction of a Prognostic Model

A total of 826 samples in GSE cohort were randomly divided into training group and test group at a ratio of 8 : 2 for 100 times. The most ideal training group and test group were selected under two conditions: (1) similar proportion of gender and survival status in two groups; (2) close number of binary classification samples after clustering expression profiles. Finally, 659 samples in the training group and 165 samples in the test group were confirmed, and no statistical difference was observed between the two groups (*Chi*-square test, *p* > 0.05, Supplementary Table S3). TCGA-STAD dataset was an independent validation group.

Survival R package of “coxph function” was conducted for univariate Cox regression analysis in the training group. Differentially expressed genes with coefficients were screened under *p* < 0.05. Least absolute shrinkage and selection operator (LASSO) regression analysis in the glmnet package [44] and step Akaike information criterion (stepAIC) in the MASS package [45] were employed to optimize the prognostic model defined as: risk score = gene 1 expression *∗* coefficient 1 + gene 2 expression *∗* coefficient 2 + … + gene *n* expression *∗* coefficient *n*. Risk score was converted to *z*-score, and *z*-score = 0 was the cut-off for stratifying samples into high-risk and low-risk groups. Receiver operating characteristic (ROC) curve and Kaplan-Meier survival curve were used to assess the prognostic model.

### 2.8. Statistics Analysis

All the statistics analyses were performed in R (v3.6.2). *p* < 0.05 was considered as a statistical significance. All statistics methods were shown in figure legends.

## 3. Results

### 3.1. Identification of Marker Genes Related to CD8 T Cells

**Figure 2:**
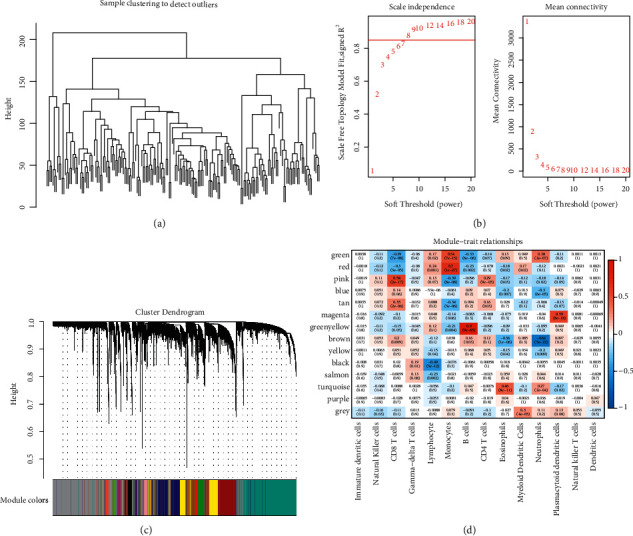
WGCNA for identifying marker genes related to CD8 T cells from immune cell datasets. (a) Hierarchical clustering tree based on 179 expression profiles in immune cells datasets. (b) Confirmation of soft threshold (power) by scale independence and mean connectivity. (c) Identification of 14 gene modules with different colors from clustering dendrogram. Grey represents gene clusters that cannot merge with others. (d) Pearson correlation rank analysis between 14 gene modules and 14 types of immune cells.

We first extracted marker genes associated with CD8 T cells. To this end, WGCNA was used to analyze expression profiles of immune cells and identify coexpressed gene modules. Hierarchical clustering analysis classified a number of immune-related genes into various branches (Figure 2(a)). For ensuring a scale-free topology nature, the Pearson correlation coefficient between log (*k*) and log (*p*(*k*)) should reach 0.85. Therefore, *β* = 8 where *β* represents power of soft threshold selected (Figure 2(b)). Based on the soft threshold and correlation coefficient between genes, a topological overlap matrix was built, and a series of gene modules were identified. Finally, after merging adjacent modules according to eigengenes, 14 coexpressed gene modules were determined (Figure 2(c)). These 14 gene modules were differently associated with various types of immune cells; here, pink module with 446 genes was found to be closely associated with CD8 T cells (coefficient = 0.58, *p*=2*e* − 17, Figure 2(d)).

**Figure 3:**
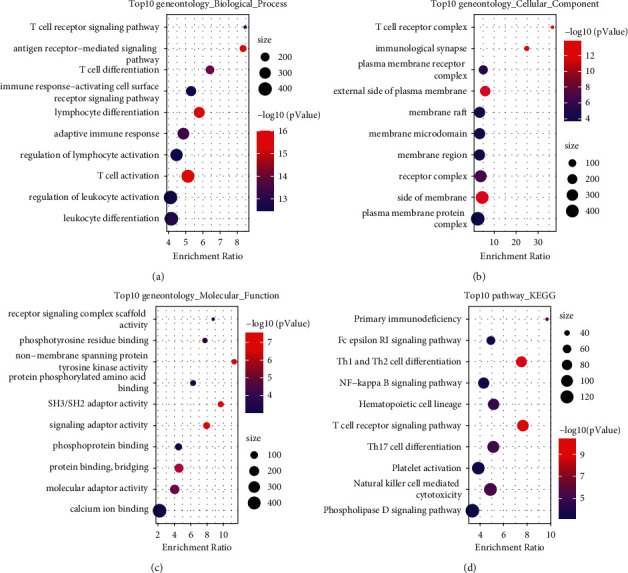
Go function and KEGG analysis of 446 genes related to CD8 T cells. (a–d) The top 10 enriched terms annotated in biological process (a), cellular component (b), molecular function (c), and KEGG pathways (d). Size means the enriched gene numbers.

KEGG and GO analysis on 446 CD8 T cells-related genes demonstrated a strong relation between these genes and immune function. The number of annotated terms of biological process, cellular component, and molecular function were 284, 46, and 26 (*p* < 0.05), respectively, and the top 10 terms were listed (Figures 3(a)–3(c)). These genes were closely involved in T cell receptor signaling pathway, antigen receptor-mediated signaling pathway, T cell differentiation, immune response-activating cell surface receptor signaling pathway, lymphocyte differentiation, etc. KEGG analysis annotated 33 pathways significantly correlated with these genes including multiple immune-related pathways, such as primary immunodeficiency, Th1 and Th2 cell differentiation, T cell receptor signaling pathway, Th17 cell differentiation, and natural killer cell mediated cytotoxicity (Figure 3(d)).

### 3.2. Construction of CD8 T Cells-Related Immune Subtypes

**Figure 4:**
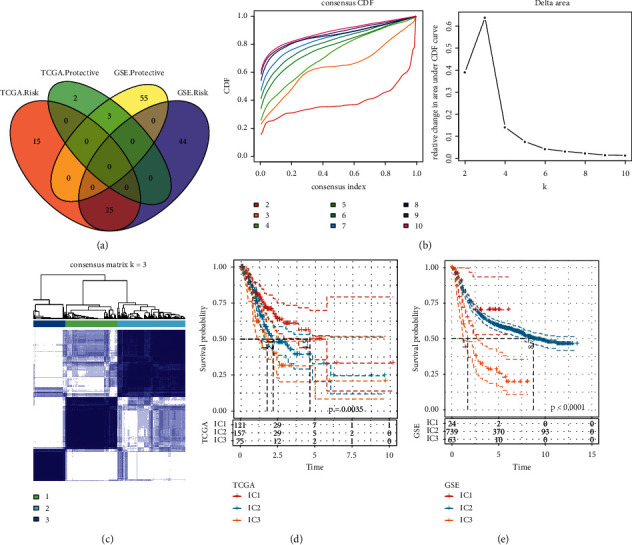
Construction of an immune subtyping system. (a) The Venn plot of genes related to prognosis screened from TCGA-STAD dataset and GSE cohort. Risk represents the negative correlation between gene expression and prognosis. Protective represents the positive correlation between them. (b) Cluster numbers *k* from 2 to 10 were analyzed to select the optimal number according to CDF and relative change in area under CDF curve. (c) The consensus matrix when *k* = 3. (d, e) Kaplan-Meier survival curve of three immune subtypes in TCGA-STAD dataset (d) and GSE cohort (e). Log-rank test was performed.

After 446 marker genes of CD8 T cells were extracted, CD8 T cells-related immune subtypes were constructed. By using univariate Cox regression analysis, 45 and 127 genes associated with GC prognosis were identified from TCGA-STAD dataset and GSE cohort, respectively. The intersection of two sets displayed a total of 28 genes, with 3 genes positively correlated with overall survival (OS) and 25 genes related to a worse OS (*p* < 0.05, Figure 4(a)). According to the expression of 28 genes, we conducted consensus clustering on 353 samples from TCGA-STAD dataset. CDF curve showed the highest relative change in area under CDF curve when cluster number *k* = 3, suggesting that the optimal cluster number was 3 (Figure 4(b), Supplementary Table S4). Consensus matrix classified 353 samples into three immune subtypes of IC1, IC2, and IC3 (IC, immune cluster; Figure 4(c)). Survival analysis revealed the distinct OS among the three subtypes with the optimal OS in IC1 and the worst OS in IC3 (*p*=0.035, Figure 4(d)). Likewise, we observed the same results in GSE cohort (*p* < 0.0001, Figure 4(e)), indicating that this immune subtyping system was valid in different datasets.

### 3.3. The Distribution of Immune Subtypes in Clinical Features

**Figure 5:**
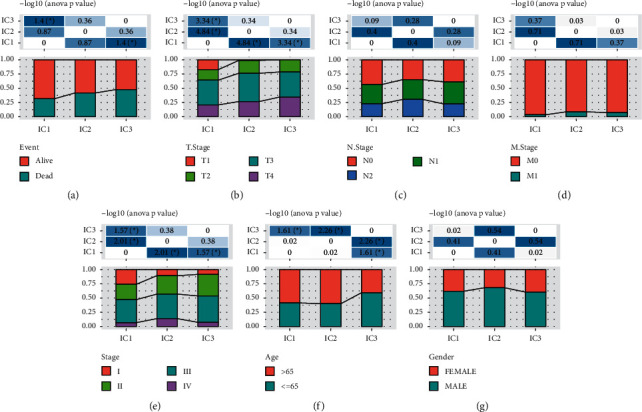
The relation between immune subtypes and clinical features, including survival status (a), T stage (b), N stage (c), M stage (d), stage I to IV (e), age (f) and gender (g).

To analyze if there was a relation between immune subtypes and clinical features, we analyzed the distribution of three subtypes in different clinical features including survival status, T stage, N stage, M stage, stage I to IV, age, and gender. The results showed that three subtypes were differentially distributed in survival status, T stage, stages I to IV, and age; however, no difference was shown in N stage, M stage, and gender (Figure 5). The proportion of deceased samples in IC3 was higher than IC1 (*p* < 0.05, Figure 5(a)), which was consistent with the worse OS of IC3. As for T stage, IC1 had the highest proportion of T1, while IC3 had the highest proportion of T4 *p* < 0.05, Figure 5(b)), showing that T stage was tightly correlated with immune subtypes. The proportion of stage I from IC1 to IC3 was decreasing (*p* < 0.05, Figure 5(e)), which may be one of the reasons contributing to the optimal prognosis of IC1 and the worst prognosis of IC3. Interestingly, age ≤65 consisted of the majority in IC3, which was opposite to IC1 and IC2 (*p* < 0.05, Figure 5(f)).

### 3.4. The Correlation between Immune Subtypes and Tumor Mutation Burden

**Figure 6:**
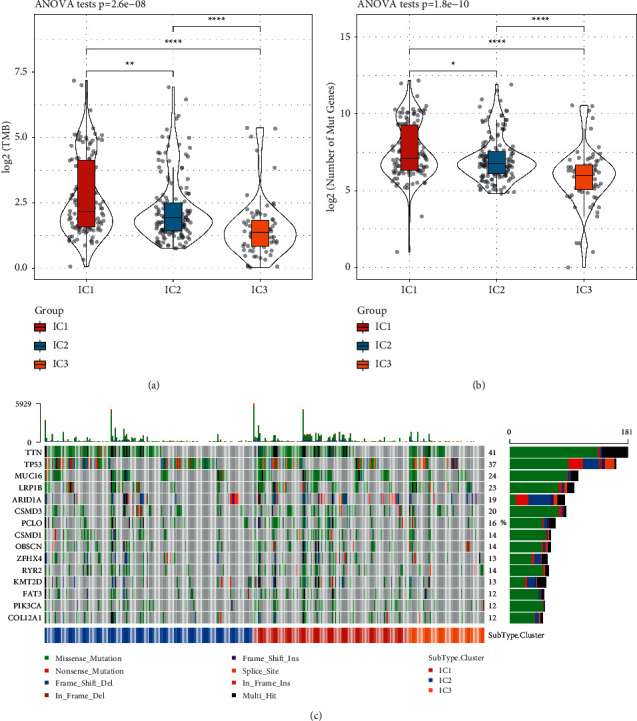
TMB and mutation patterns in TCGA-STAD dataset. ((a) and (b)) TMB (a) and number of mutated genes (b) of three immune subtypes. ANOVA was performed. (c) Mutation patterns of the top 15 genes in three immune subtypes. 8 types of mutations were presented including missense, nonsense, frame-shift deletions or insertions, in-frame deletions or insertions, splice site mutations, and different combinations of multiple genetic mutations (multi-hit).  ^*∗*^*p* < 0.05,  ^*∗∗*^*p* < 0.01,  ^*∗∗∗∗*^*p* < 0.0001.

We calculated the tumor mutation burden (TMB) of each sample in TCGA-STAD dataset using mutect2 software. Distinct TMB was shown in three immune subtypes, with the highest TMB in IC1 and the lowest TMB in IC3 (*p*=2.6*e* − 8, Figure 6(a)). Consistently, IC1 had the most numbers of mutated genes, while IC3 had the least (*p*=1.8*e* − 10, Figure 6(b)). Furthermore, 10031 genes were screened with a mutation frequency up to 3%; here, 1636 genes were found to be significantly mutated using *Chi*-square test (*p* < 0.05). The mutation patterns of the top 15 mutated genes were displayed in Figure 6(c). The proportion of TP53 mutations accounted for 37%, and other highly mutated genes such as MUC16, LRP1B, and ARID1A were reported to be closely associated with various cancers.

### 3.5. Differential Expression of Chemokines and Immune Checkpoints among Immune Subtypes

**Figure 7:**
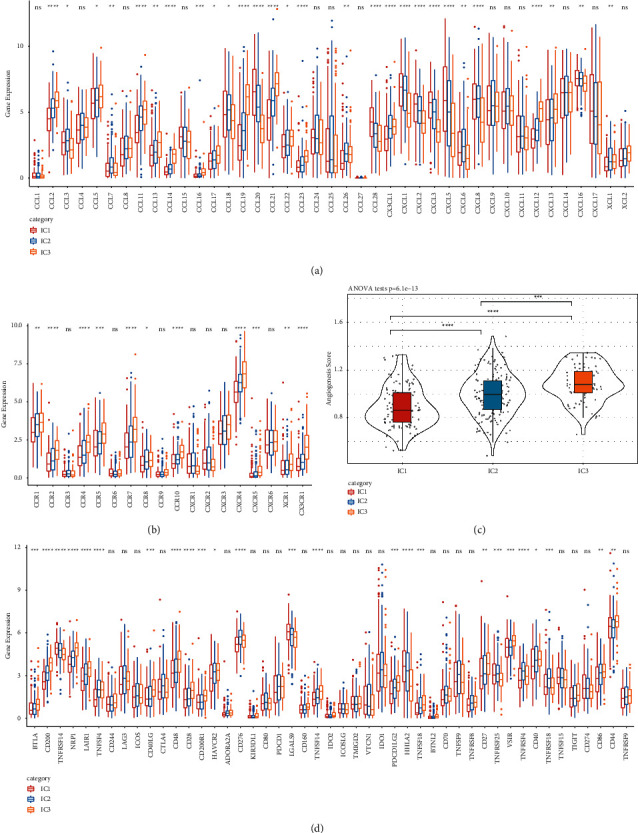
Expression of chemokines, chemokine receptors and genes related to immune checkpoints in TCGA-STAD dataset. ((a) and (b)) Expression of 41 chemokines (a) and 18 chemokine receptors (b) in three subtypes. (c) Differential angiogenesis score among three subtypes. (d) Expression of 47 genes related to immune checkpoints in three subtypes. ANOVA was performed.  ^*∗*^*p* < 0.05,  ^*∗∗*^*p* < 0.01,  ^*∗∗*^*p* < 0.001,  ^*∗∗∗∗*^*p* < 0.0001.

Chemokines play a pivotal role in determining TME by recruiting and orchestrating immune cells, which can elicit or inhibit antitumoral responses. Through binding with chemokine receptors, chemokines promote tumor proliferation, tumor angiogenesis, and migration. Therefore, we assessed the expression of 41 chemokines and 18 chemokine receptors of three immune subtypes and observed that 28 out of 41 chemokines and 11 out of 18 chemokines receptors were differentially expressed among the three subtypes, and that the majority of them were higher-expressed in IC3 (Figures 7(a) and 7(b)), which may lead to a distinct TME. As chemokines are critical for tumor angiogenesis that is necessary for tumor proliferation and migration, we also evaluated the angiogenesis score of each sample in TCGA-STAD dataset according to a series of genes related to angiogenesis [46]. Significant difference was observed among three subtypes that the angiogenesis score was the lowest in IC1 but the highest in IC3, which was consistent with their prognosis (Figures 7(c) and 4(d)). Immune checkpoints are responsible for transducing signals between immune cells; thereby, they can regulate cytokine secretion in response to TME. We obtained 47 genes related to immune checkpoints from previous research [47] and analyzed their expression of each sample. The result showed that 25 out of 47 genes were differentially expressed among IC1, IC2, and IC3 (Figure 7(d)), suggesting that these 25 genes related to immune checkpoints were closely involved in contributing to different TMEs.

### 3.6. Differential Enrichment of Immune Cells and Oncogenic Pathways among Immune Subtypes

**Figure 8:**
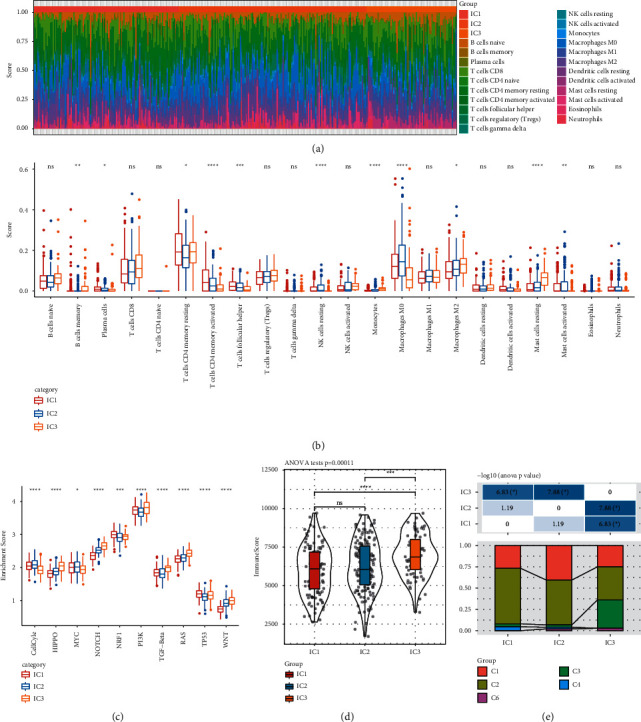
Immune features of three immune subtypes in TCGA-STAD dataset. (a) The heatmap presenting the distribution of 22 immune cells. (b) Comparison of enrichment score of 22 immune cells among three subtypes. (c) The enrichment of 10 oncogenic pathways in three subtypes. (d) Total immune score of three subtypes. (e) The distribution of pan-cancer immune subtypes in three subtypes. ANOVA was performed. ns, no significance.  ^*∗*^*p* < 0.05,  ^*∗∗*^*p* < 0.01,  ^*∗∗*^*p* < 0.001,  ^*∗∗∗∗*^*p* < 0.0001.

As the expression of chemokines and genes related to immune checkpoints varied in three immune subtypes, we further analyzed the distribution of immune cells and activity of tumor-related pathways. CIBERSORT was employed to calculate enrichment score of 22 types of immune cells. Among these immune cells, CD8 T cells, resting memory CD4 T cells, M0 macrophages, and M2 macrophages were apparently higher enriched than others, and 8 immune cells were differentially enriched in three subtypes, including naive B cells, activated memory CD4 T cells, helper follicular T cells, resting NK cells, monocytes, M0 macrophages, M2 macrophages, and resting dendritic cells (Figures 8(a) and 8(b)). Activated memory CD4 T cells were highly enriched in IC1, enabling more active antitumor response, although no difference of enrichment of CD8 T cells was observed in the three subtypes. A low proportion of M0 macrophages and a high proportion of M2 macrophages were found in IC3, which could explain the increased number of tumor-associated macrophages (TAMs). IC3 had the highest immune score than IC1 and IC2, which may result from a high expression of chemokines and chemokine receptors in IC3 (Figures 8(d), 7(a) and 8(b)).

In addition, we evaluated the enrichment of 10 oncogenic pathways in the three subtypes [48], and all pathways were differentially enriched in the three subtypes (Figure 8(c)). Noticeably, IC3 was significantly higher-enriched than IC1 and IC2 in the most pathways, including Hippo signaling pathway, Notch signaling pathway, PI3K signaling pathway, TGF-*β* signaling pathway, RAS signaling pathway, and WNT signaling pathway (*p* < 0.0001, Figure 8(c)).

According to various aspects of analysis, the three immune subtypes presented significant difference and correlation in prognosis, TME, and oncogenic pathways, demonstrating the effectiveness of this immune subtyping system. Compared with the previous immune subtypes in a pan-cancer research [7], a close relation was also discovered. The pan-cancer research divided gastric cancer into five immune subtypes (C1, C2, C3, C4, and C6) with different OS, and the distribution of five subtypes was assessed in IC1, IC2, and IC3 (Figure 8(e)). C2 subtype with favorable OS consisted of a high proportion of IC1 and a low proportion of IC3. C3 subtype with worse OS than C2 was densely gathered in IC3, and C6 subtype with the worst OS only presented in IC1 and IC2 (Figure 8(e) and Supplementary Figure S3). The results further proved that our immune subtyping system was solid and reliable in predicting gastric cancer prognosis.

### 3.7. Immune Escape and T Cell Function Analyzed by TIDE

**Figure 9:**
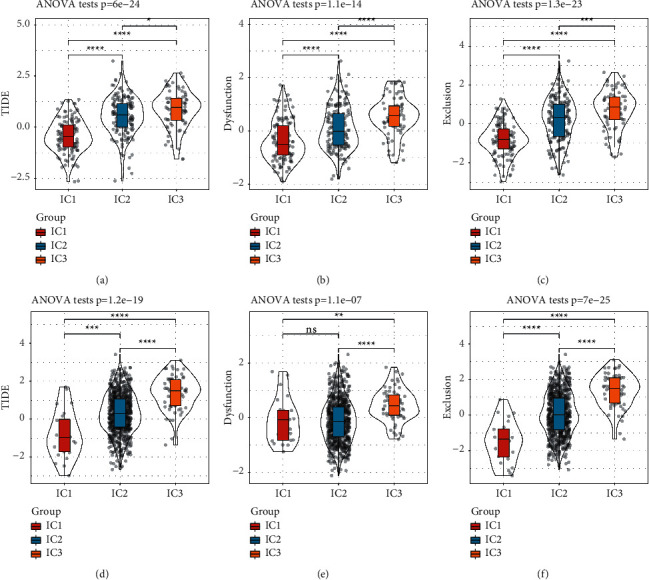
TIDE analysis for predicting the efficacy of immunotherapy in different subtypes. (a–c) TIDE score (a), T cell dysfunction score (b), and T cell exclusion score (c) of samples in TCGA-STAD dataset. (d–f) TIDE score (d), T cell dysfunction score (e), and T cell exclusion score (f) of samples in GSE cohort. ANOVA was performed. ns, no significance.  ^*∗*^*p* < 0.05,  ^*∗∗*^*p* < 0.01,  ^*∗∗*^*p* < 0.001,  ^*∗∗∗∗*^*p* < 0.0001.

Next, we analyzed whether there was a difference among IC1, IC2, and IC3 on their immune response using TIDE methodology [49]. In TCGA-STAD dataset, IC1 had the lowest TIDE score, and IC3 had the highest (Figure 9(a)), indicating a high possibility of immune escape in IC1. The function of T cells is an important factor that can directly affect the immune response against tumor cells. Therefore, we also analyzed the manifestation of T cell function from the aspects of dysfunction and exclusion. IC1 showed the lowest score of both T cell dysfunction and exclusion, while IC3 had the highest score of the two (Figures 9(b) and 9(c)), suggesting impaired function of T cells to kill tumor cells in IC3. The similar results were also found in GSE cohort (Figures 9(d)–9(f)).

### 3.8. Differential Sensitivity of Three Immune Subtypes to PD-1 Inhibitor and Chemotherapeutic Drugs

**Figure 10:**
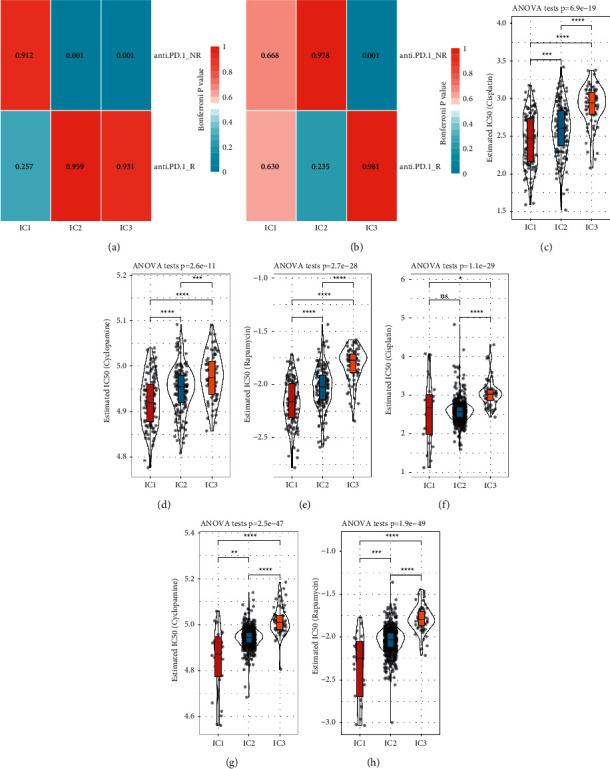
Different responses to immunotherapy and chemotherapy among three immune subtypes. ((a, b)) Submap analysis between GSE78220 and TCGA-STAD dataset (a), GSE78220 and GSE cohort (b). Bonferroni correction was applied to correct (*p*) value. Anti-PD-1_NR and anti-PD-1_R groups represent nonresponsive and responsive to anti-PD-1 therapy respectively. (c–e) Estimated IC50 of cisplatin (c), cyclopamine (d) and rapamycin (e) in TCGA-STAD dataset. (f–h) Estimated IC50 of cisplatin (f), cyclopamine (g) and rapamycin (h) in GSE cohort. ANOVA was performed. ns, no significance.  ^*∗*^*p* < 0.05,  ^*∗∗*^*p* < 0.01,  ^*∗∗*^*p* < 0.001,  ^*∗∗∗∗*^*p* < 0.0001.

Anti-PD-1/PD-L1 therapy using PD-1/PD-L1 inhibitors to active or reactive immune response to tumor cells is one of the most promising immunotherapies for treating many cancer types. We performed submap analysis to compare the similarity of TME between samples treated by anti-PD-1 inhibitor in GSE78220 dataset and three immune subtypes. High similarity with a low *p* value indicated a high efficacy of anti-PD-1 therapy. IC3 was shown to be not sensitive to anti-PD-1 therapy in both TCGA-STAD dataset and GSE cohort (Bonferroni-corrected *p*=0.001, Figures 10(a) and 10(b)). However, IC1 and IC2 showed different responses to anti-PD-1 therapy in two datasets. Furthermore, we also examined the response to chemotherapeutic drugs by calculating estimated IC50. Lower IC50 was indicative of a higher drug sensitivity and possibly a more favorable outcome. In TCGA-STAD dataset, IC1 displayed the lowest estimated IC50 of all three drugs (cisplatin, cyclopamine, and rapamycin), indicating that IC1 had the highest sensitivity to these drugs (Figures 10(c)–10(e)); however, IC3 could only limitedly benefit from the treatment of these drugs. Simultaneously, consistent results were observed in GSE cohort (Figures 10(f)–10(h)).

### 3.9. Construction of a Prognostic Model Based on Marker Genes Related to CD8 T Cells

Although the immune subtyping system can stratify GC patients into three subtypes with distinct prognosis and can largely guide chemotherapy and immunotherapy, it is not effective in predicting the treatment outcomes of GC patients. Based on the genes related to CD8 T cells, we constructed a prognostic model with the least number of genes to simply and efficiently predict prognosis. To this end, GSE cohort was randomly divided into training group and test group (Supplementary Table S3), with TCGA-STAD dataset as an independent validation group.

Within the training group, we screened 107 differentially expressed genes related to OS using univariate Cox regression analysis (*p* < 0.05). Then, LASSO regression analysis was conducted to compress the model and reduce number of genes. The coefficient of each gene was close to zero with the increasing value of lambda (Supplementary Figure S4A). 10-fold cross validation was applied to construct model with different lambda, and the confidential interval of different lambda was calculated (Supplementary Figure S4B). When lambda = 0.0671, the optimal model consisting a total of 12 genes was developed (Supplementary Figure S4). Then, we applied stepAIC to further optimize the model, and finally an 8-gene prognostic model was constructed as follows:

(1) $$\begin{array}{c} \text{Risk score}=0.358*\text{FBLN}5+0.307*\text{ENPP}5-0.665*\text{KLHDC}4-0.620*\text{CD}160+0.890*\text{ZNF}578+0.751*\text{LBH}-0.864*\text{KLRD}1+0.215*\text{TCEAL}2. \end{array}$$

**Figure 11:**
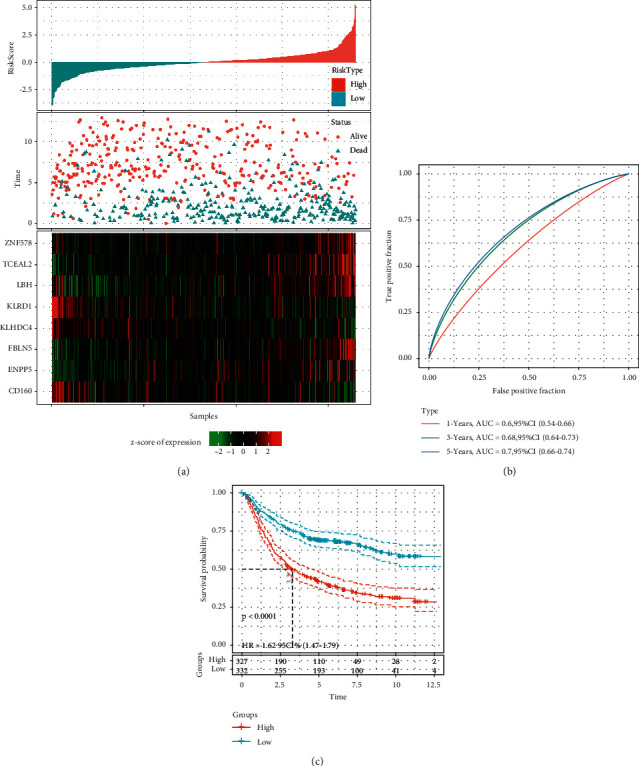
Assessment of the prognostic model in the training group. (a) The distribution of high-risk and low-risk groups, and the expression of 8 prognostic genes corresponding to risk score. (b) ROC analysis and AUC of 1-year, 3-year and 5-year OS predicted by the 8-gene signature. (c) Kaplan-Meier survival curve of high-risk and low-risk groups. Log-rank test was performed.

The risk score of each sample was counted using the 8-gene signature, and risk score was converted to z-score. Each sample was classified into low-risk and high-risk groups by the cutting of z-score = 0. In the training group, 327 samples and 332 samples were classified into high-risk and low-risk groups, respectively, with the high-risk group showing more deceased samples (Figure 11(a)). ZNF578, TCEAL2, LBH, FBLN5, and ENPP5 were highly expressed in high-risk group, while KLHDC4, KLRD1, and CD160 were low-expressed in low-risk group (Figure 11(a)). ROC analysis manifested the reliability of the classification that AUC of 1-year, 3-year, and 5-year was 0.60, 0.68, and 0.70, respectively (Figure 11(b)). Survival curve revealed the significantly distinct OS between two groups, with a better prognosis in low-risk group (*p* < 0.0001, Figure 11(c)). Risk score could be an independent factor to efficiently predict prognosis (HR = 1.62, 95% CI = 1.47–1.79, Figure 11(c)). We therefore assessed the prognostic model in the test group. 165 samples were stratified into high-risk and low-risk groups with distinct OS (*p* < 0.0001, Supplementary Figure S5). The robustness of the prognostic model was also validated in TCGA-STAD dataset, and 353 samples were classified into low-risk and high-risk groups with differential OS (*p*=0.002, Supplementary Figure S6). In addition, we also analyzed the expression differences of these eight genes between cancer and adjacent samples. We can observe that most of these genes have significant expression differences, such as KLHDC4, ZNF578, LBH, and KLRD1 that are significantly overexpressed in tumor samples and tceal2 that is significantly underexpressed in adjacent samples (Supplementary Figure S7A). Further, we observed the expression differences of these genes in three molecular subtypes; FBLN5, LBH, and TCEAL2 were specifically highly expressed in IC3, and KLHDC4 was specifically low expressed in IC3 (Supplementary Figure S7B). The above results indicated that the 8-gene signature was effective in GC prognosis prediction.

### 3.10. Risk Score was Associated with Clinical Features and Immune Subtypes

**Figure 12:**
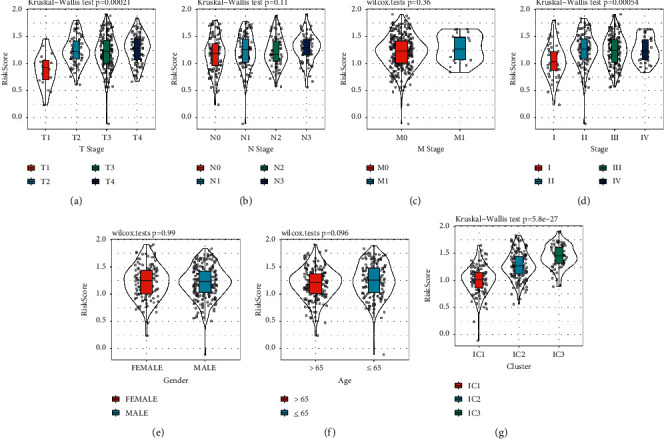
The relation of risk score to T stage (a), N stage (b), M stage (c), stage I to IV (d), gender (e), age (f) and immune subtypes (g). Wilcoxon test was performed.

**Figure 13:**
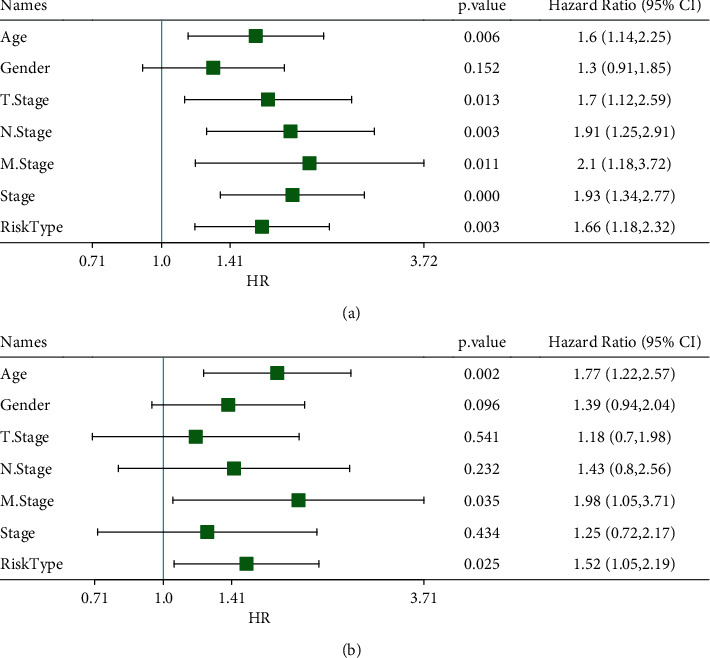
Univariate (a) and multivariate (b) Cox regression analysis between potential risk factors and prognosis.

Then, we analyzed the relation between risk score and clinical features including T, N, M stage, stages I to IV, gender, and age and found that low risk score was presented in T1, N0, M0 stage, and stage I with clinically mild progression (Figures 12(a)–12(d)). Especially, significantly differential risk score was distributed in T stage (*p*=0.00021), stages I to IV (*p*=0.00054). However, gender and age were not the factors affecting risk score (Figures 12(e) and 12(f)). Notably, a strong correlation was observed between risk score and immune subtypes, where IC1 had the lowest risk score and IC3 showed the highest risk score (*p*=5.8*e* − 27, Figure 12(g)). These results further demonstrated the viability of the prognostic signature. Moreover, hazard ratio of clinical features and risk type was assessed with univariate and multivariate Cox regression analysis using TCGA-STAD dataset. Risk type was significantly associated with overall survival, with HR = 1.66 (95% CI = 1.18–2.32, *p*=0.003) and HR = 1.52 (95% CI = 1.05–2.19, *p*=0.025) in univariate and multivariate Cox regression analysis, respectively (Figure 13). Moreover, age and M stage were also risk factors, with HR >1, which could be included to delineate nomogram together with risk score.

### 3.11. The Correlation between Risk Score and KEGG Pathways

**Figure 14:**
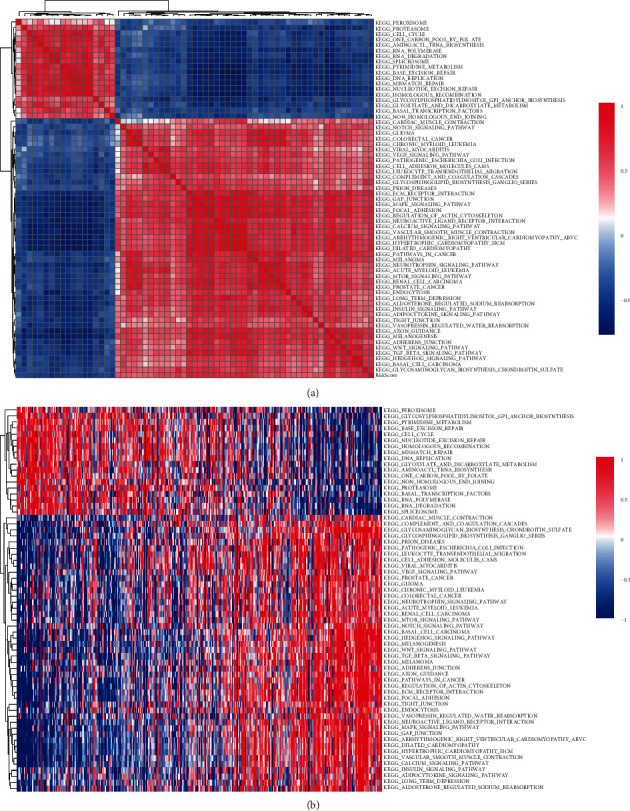
The relation between risk score and KEGG pathways. (a) 64 KEGG pathways related to risk score identified by Pearson correlation analysis. |correlation coefficient| >0.4. (b) The relation between enrichment of pathways and risk score. Horizontal axis represents the increasing risk score from left to right. Red means positive correlation and blue means negative correlation.

To further examine whether risk score and functional pathways were correlated, ssGSEA was performed to calculate enrichment score of each sample in TCGA-STAD dataset, followed by correlation analysis between enrichment score in functional pathways and risk score using Pearson correlation analysis. |Correlation coefficient| ≥0.4 was set as a cut-off to screen the functional pathways closely associated with risk score. 64 KEGG pathways, including 18 pathways negatively correlated with risk score and 46 pathways positively correlated with risk score, were identified (Figure 14(a)). Pathways related to cell cycle, DNA replication, and DNA repair were greatly enriched in the samples with low risk score, while tumor-related pathways, such as VEGF signaling pathway, NOTCH signaling pathway, TGF-*β* signaling pathway, WNT signaling pathway, and MAPK signaling pathway, were highly enriched in the samples with high risk score (Figure 14(b)). The strong correlation between high risk score and high enrichment of oncogenic pathways showed that the 8 prognostic genes may be closely involved in promoting tumor progression through activating or regulating oncogenic pathways.

### 3.12. Construction of a Nomogram to Predict Prognosis

**Figure 15:**
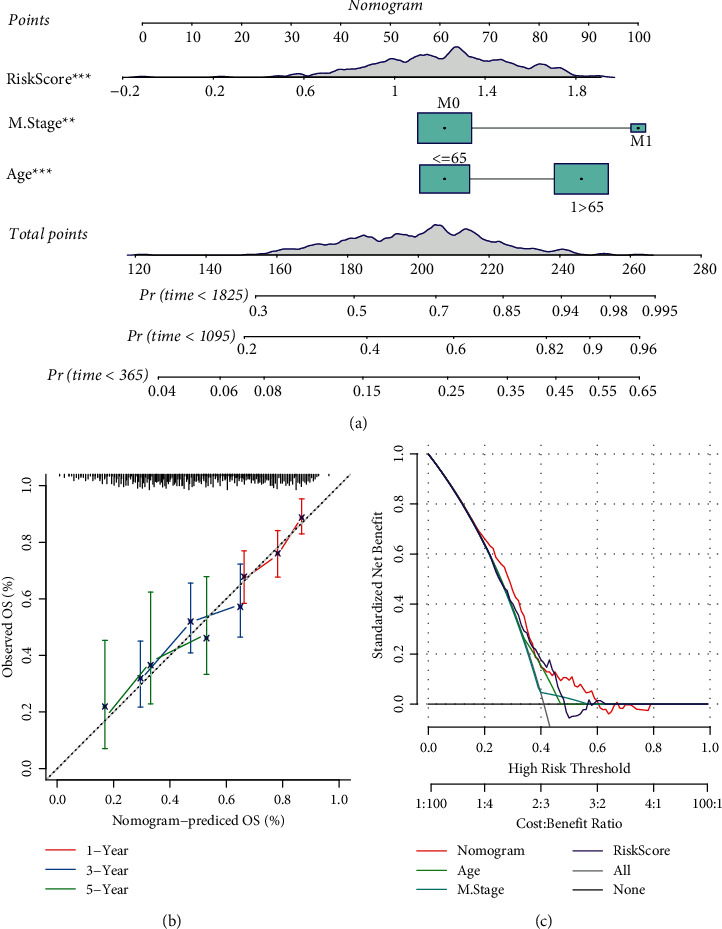
Visualization of the prognostic model. (a) A nomogram based on risk score, M stage and age for predicting overall survival. (b) The correction plot of 1-year, 3-year and 5-year OS predicted by the nomogram. (c) DCA curve of nomogram, age, M stage and risk score.

To more precisely predict overall survival, we constructed a nomogram combining three risk factors (risk score, M stage, and age). Each risk factor corresponds to a point according to clinical information, and the total points correspond to the predicted death possibility in 1-, 3-, and 5-year (Figure 15(a)). The predicted OS was corrected by the observed OS (Figure 15(b)). Decision curve analysis (DCA) was performed to evaluate the effectiveness of the nomogram. As a result, nomogram was more advantageous to predict prognosis than risk score only (Figure 15(c)).

### 3.13. Prognostic Significance of Risk Score in Immunotherapy

**Figure 16:**
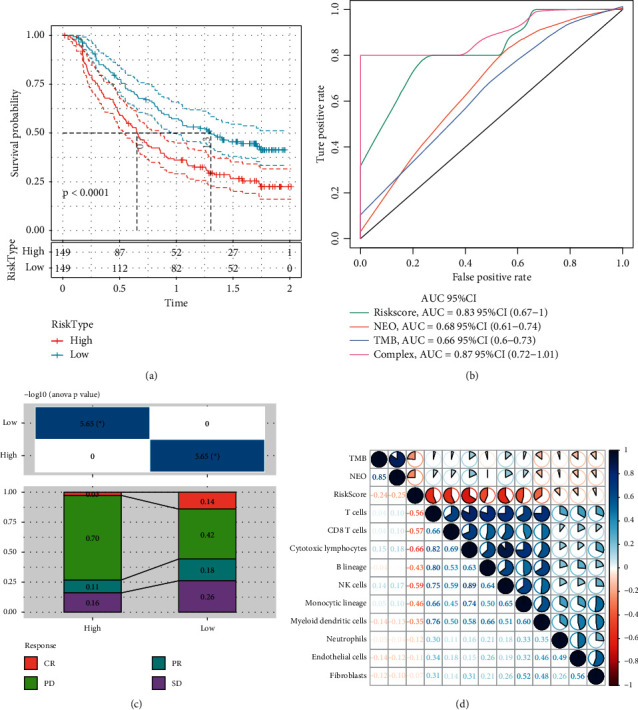
The application of the 8-gene prognostic signature in Imvigor210 dataset with patients treated by anti-PD-L1 inhibitors. (a) Kaplan-Meier survival curve of high-risk and low-risk groups. Log-rank test was performed. (b) ROC analysis of risk score, NEO and TMB in predicting prognosis. (c) The distribution of CR, PD, PR, SD patients in high-risk and low-risk groups. ANOVA was performed. (d) Pearson correlation analysis between risk score and TMB, NEO, and immune infiltration. Blue represents positive correlation and red represents negative correlation.

We further examined whether the 8-gene signature was associated with the efficacy of immunotherapy. Imvigor210 dataset containing metastatic urothelial carcinoma patients treated by anti-PD-L1 immunotherapy was used in the following analysis. Kaplan-Meier survival curve showed a more favorable OS in low-risk group (*p* < 0.0001, Figure 16(a)). In comparison to neoantigen (NEO) and TMB, risk score with an AUC of 0.83 (95% CI = 0.67–1.00, Figure 16(b)) was the most effective when predicting prognosis. Between high-risk and low-risk groups, differential responses to immunotherapy were detected, where the proportion of complete response (CR) and stable disease (SD) was found to be significantly higher in low-risk group (*p* < 0.05, Figure 16(c)). In the relation to immune infiltration, risk score was negatively related to many types of immune cells such as CD8 T cells, cytotoxic lymphocytes, B lineage, and NK cells, while NEO and TMB were not obviously correlated with these immune cells (Figure 16(d)). Moreover, lower risk score was related to higher NEO and TMB, suggesting that patients with higher NEO and TMB could benefit much more from anti-PD-L1 therapy.

**Figure 17:**
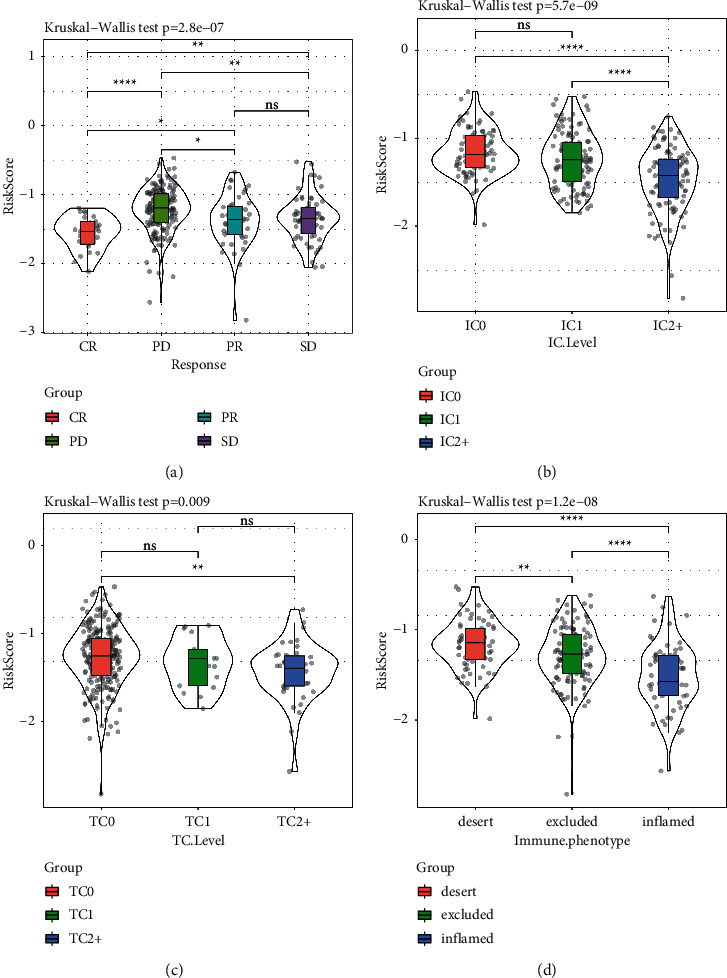
The relation of risk score to response treated by anti-PD-L1 (a), PD-L1-postive immune cells (b), PD-L1-postive tumor cells (c), and immune phenotypes (d). IC2+ represents IC2/IC3 and TC2+ represents TC2/TC3. Kruskal-Wallis test was performed. ns, no significance.  ^*∗*^*p* < 0.05,  ^*∗∗*^*p* < 0.01,  ^*∗∗*^*p* < 0.001,  ^*∗∗∗∗*^*p* < 0.0001. IC, immune cell. TC, tumor cell.

In addition, we analyzed the risk score in different kinds of groups, including treatment response, immune cells, tumor cells, and immune phenotype. CR patients had the lowest risk score among CR, PD, PR, and SD patients (Figure 17(a)). Previous study divided immune cells (IC) and tumor cells (TC) into three groups, according to the percentage of PD-L1 positive cells: IC0/TC0 (<1%), IC1/TC1 (≥1% but <5%) and IC2+/TC2+ (≥5%) [50]. The result showed that IC2+ and TC2+ group had the lowest risk score (Figures 17(b) and 17(c)), indicating that patients showing TME enriched with PD-L1-positive cells could be treated by anti-PD-L1 therapy. In terms of three immune phenotypes (desert, excluded, and inflamed), inflamed phenotype has been reported to be actively responsible to immunotherapy, which was consistent with the present result that inflamed group had the lowest risk score (*p* < 0.0001, Figure 17(d)). These analyses demonstrated that the prognostic signature was robust to predict outcomes for patients who have undergone immunotherapy.

## 4. Discussion

The antitumor effects of cytotoxic CD8 T cells rely on CD8 T cell differentiation and its infiltration in tumor site but can be suppressed by cytokines and chemokines secreted from tumor cells and immune cells in TME. It has been demonstrated that the inhibition of PD-1/PD-L1 can activate the function of cytotoxic CD8 T cells, thereby suppressing tumor proliferation. However, the anti-PD-1/PD-L1 therapy is only effective to certain cancer patients due to differential TME of patients. Therefore, an effective molecular subtyping system is strongly needed to characterize TME and status of CD8 T cells, so as to predict the outcomes of immunotherapy. Although previous studies have developed various types of molecular subtypes for gastric cancer [7, 51], none of them focuses on CD8 T cells. In the present study, we constructed three immune subtypes (IC1, IC2, and IC3) based on marker genes related to CD8 T cells and fully characterized the tight relation among immune subtypes, TME, oncogenic pathways, chemotherapy, and immunotherapy.

Chemokines play a critical role in facilitating the migration of immune cells to tumor site and can also modulate tumor cell metastasis and growth [52]. Differential expression of chemokines and chemokine receptors was shown in three immune subtypes, which may explain the distinct OS outcomes of the three. CCL2, CCL5, CCL17, and CCL22 can induce immunosuppressive cell migration through their interactions with their receptors of CCR2, CCR5, and CCR4 in macrophages and regulatory T cells [53]. High expression of CCL2, CCL5, CCL17, and CCL22 and their receptors were observed in IC3 (Figure 7), which was related to a poor prognosis of IC3. CXCR4 can direct the migration of CD8 T cells and NK cells to tumor sites [54] but can also impede the infiltration of T cells to tumor cells through CXCL12 [55]. Pharmacological studies targeting CXCR4/CXCL12 axis demonstrated that CXCR4 antagonist releases T cells from CXCL12-rich stroma and increases T cell infiltration to tumor sites [55–57]. Moreover, CXCL12 can induce epithelial-mesenchymal transition (EMT) and gastric cancer metastasis possibly through the interaction between MET proto-oncogene (c-MET) and CXCR4 [58]. Among three immune subtypes, the expression tendency of CXCL12 was corresponding with CXCR4 expression, and IC3 had the highest expression level of them, which was consistent with its poor outcome. In addition, previous studies discovered that low expression of CXCL8 is associated with unfavorable prognosis in gastric cancer [59, 60], and the same phenomenon is also observed in the present study. These observations proved that our immune subtyping system based on CD8 T cells was reliable.

Immune checkpoint blockade, such as inhibiting PD-1/PD-L1 axis, is a promising immunotherapy for the management of metastatic cancer patients. PD-1 expressed by CD8 T cells can interact with its ligand PD-L1 expressed by immune cells or tumor cells in TME, leading to T cell exhaustion and apoptosis, which refers to immune escape [61]. TIDE analysis revealed that IC3 had the highest score of T cell exhaustion and exclusion, indicating its impaired T cell function and poor prognosis (Figure 9). High expression of CXCL12 and CXCR4 was the possible reason for promoting the interaction between PD-1 and PD-L1, further triggering T cell dysfunction. In other words, high immune escape score of IC3 probably resulted from the activation of CXCL12/CXCR4 and PD-1/PD-L1 axis. Various immune checkpoint inhibitors have been examined in cancer patients; however, only around 20% of patients can obtain long-term benefits [3]. Our immune subtyping system could guide a better personalized therapy to GC patients.

The three immune subtypes manifested differential enrichment in oncogenic pathways, especially cell cycle, HIPPO, NOTCH, PI3K, TGF-*β*, RAS, and WNT signaling pathways. Apart from cell cycle pathway, activation of remained pathways is closely related to poor prognosis of cancer patients. Some inhibitors targeting HIPPO, NOTCH, PI3K, TGF-*β*, and WNT signaling pathways have been applied in clinical trials [62–65]. High enrichment of PI3K signaling pathway in GC samples, especially in IC3, may be a potential target for effective targeted drug therapy for GC patients. As for chemotherapeutic drugs, the subtyping system can also provide a direction for their clinical use; here, IC1 was found to be the most sensitive to cisplatin, cyclopamine, and rapamycin (Figures 10(c)–10(h)).

To further evaluate the clinical outcomes of GC patients, we developed an 8-gene prognostic signature and constructed a nomogram with an easy application in clinical practice. The signature can calculate the risk score of each patient and clearly stratify the patients into high-risk and low-risk groups with distinctly different prognosis. Functional analysis demonstrated that the risk score was closely associated with oncogenic pathways, such as cell cycle, NOTCH, WNT, and TGF-*β* signaling pathways (Figure 14). Furthermore, the signature also exhibited robust performance in screening metastatic urothelial carcinoma patients treated by anti-PD-1 (Figures 16(a)–16(c)). As for the relation between risk score and immune infiltration, high infiltration of CD8 T cells, cytotoxic lymphocytes, and NK cells is negatively correlated with risk score (Figure 16(d)), suggesting that immune infiltration was a critical factor of prognosis of patients who received anti-PD-1 therapy. Consistent with previous studies, in this study, patients with immune-desert phenotype had poor outcome of immunotherapy, while those with immune-inflamed phenotype can benefit much from immunotherapy [66] (Figure 17(d)).

By an integrated analysis on functional pathways, TME, immune response, immunotherapy, etc., we comprehensively characterized the links among them and demonstrated the reliability of the immune subtyping system. This subtyping system based on CD8 T cells together with the prognostic signature has demonstrated its applicability in clinical practice.

## 5. Conclusion

In conclusion, based on genes related to CD8 T cells, we developed three immune subtypes and an 8-gene prognostic signature to guide personalized therapy for GC patients. Three immune subtypes manifested differential responses to chemotherapy and immunotherapy. The prognostic signature can predict whether GC patients can benefit from immunotherapy.

## Data Availability

The data used to support the findings of this study are included within the article.

## Abbreviations

AIC: Akaike information criterion
AUC: Area under ROC curve
CDF: Cumulative distribution function
c-MET: MET proto-oncogene
CR: Complete response
DCA: Decision curve analysis
EMT: Epithelial-mesenchymal transition
GC: Gastric cancer
GEO: Gene expression omnibus
GO: Gene ontology
HR: Hazard ratio
IC: Immune cluster
IC50: The biochemical half maximal inhibitory concentration
KEGG: Kyoto encyclopedia of genes and genomes
LASSO: Least absolute shrinkage and selection operator
NEO: Neoantigen
OS: Overall survival
PCA: Principle component analysis
PD: Progressive disease
PD-1: Programmed cell death protein 1
PD-L1: Programmed cell death ligand 1
PR: Partial response
ROC: Receiver operating characteristic
SD: Stable disease
SEER: The surveillance, epidemiology, and end results
ssGSEA: Single sample gene set enrichment analysis
TAMs: Tumor-associated macrophages
TCGA: The cancer genome atlas
TMB: Tumor mutation burden
TME: Tumor microenvironment
TOM: Topological overlap matrix
WGCNA: Weighted correlation network analysis.

## Supplementary Materials

Supplementary Materials Supplementary Figure S1. Elimination of batch effects for GSE cohort. (A) The PCA result before eliminating batch effects. (B) The PCA result after eliminating batch effects. Supplementary Figure S2. Elimination of batch effects for immune cell datasets. (A) The PCA result before eliminating batch effects. (B) The PCA result after eliminating batch effects. Supplementary Figure S3. Kaplan-Meier survival curve of C1, C2, C3, C4, and C6 immune subtypes reported in the previous study. Log-rank test was performed. Supplementary Figure S4. LASSO regression analysis for optimizing the model. (A) The coefficients of each variate (gene) change with different lambda values. The red dotted line represents when lambda = 0.0671, the model was the optimal. (B) Confidential interval under different lambda values. The red dot represents the site of the optimal lambda. Supplementary Figure S5. Assessment of the prognostic model in the test group. (A) The distribution of high-risk and low-risk groups, and the expression of 8 prognostic genes corresponding to risk score. (B) ROC analysis and AUC of 1-year, 3-year and 5-year OS predicted by the 8-gene signature. (C) Kaplan-Meier survival curve of high-risk and low-risk groups. Log-rank test was performed. Supplementary Figure S6. Validation of the prognostic model in TCGA-STAD dataset. (A) The distribution of high-risk and low-risk groups, and the expression of 8 prognostic genes corresponding to risk score. (B) ROC analysis and AUC of 1-year, 3-year and 5-year OS predicted by the 8-gene signature. (C) Kaplan-Meier survival curve of high-risk and low-risk groups. Log-rank test was performed. Supplementary Figure S7. The expression difference of 8 genes. (A) Expression difference of 8 genes in cancer and adjacent cancer. (B) Expression difference of 8 genes in three molecular subtypes. Supplementary Table S1. Sample information of each data set. Supplementary Table S2. The clinical information of TCGA-STAD dataset and GSE cohort. Supplementary Table S3. The information of training group and test group. Supplementary Table S4. Molecular subtypes of TCGA samples.
