# Supplementary material for: Correlation between Tumor Microenvironment and Immune Subtypes Based on CD8 T Cells Enhancing Personalized Therapy of Gastric Cancer
Source: J Oncol. 2022 Feb 28;2022:8933167. doi: 10.1155/2022/8933167 (PMC8901316; doi:10.1155/2022/8933167)
Supplement: Supplementary Materials — Supplementary Figure S1. Elimination of batch effects for GSE cohort. (A) The PCA result before eliminating batch effects. (B) The PCA result after eliminating batch effects. Supplementary Figure S2. Elimination of batch effects for immune cell datasets. (A) The PCA result before eliminating batch effects. (B) The PCA result after eliminating batch effects. Supplementary Figure S3. Kaplan-Meier survival curve of C1, C2, C3, C4, and C6 immune subtypes reported in the previous study. Log-rank test was performed. Supplementary Figure S4. LASSO regression analysis for optimizing the model. (A) The coefficients of each variate (gene) change with different lambda values. The red dotted line represents when lambda = 0.0671, the model was the optimal. (B) Confidential interval under different lambda values. The red dot represents the site of the optimal lambda. Supplementary Figure S5. Assessment of the prognostic model in the test group. (A) The distribution of high-risk and low-risk groups, and the expression of 8 prognostic genes corresponding to risk score. (B) ROC analysis and AUC of 1-year, 3-year and 5-year OS predicted by the 8-gene signature. (C) Kaplan-Meier survival curve of high-risk and low-risk groups. Log-rank test was performed. Supplementary Figure S6. Validation of the prognostic model in TCGA-STAD dataset. (A) The distribution of high-risk and low-risk groups, and the expression of 8 prognostic genes corresponding to risk score. (B) ROC analysis and AUC of 1-year, 3-year and 5-year OS predicted by the 8-gene signature. (C) Kaplan-Meier survival curve of high-risk and low-risk groups. Log-rank test was performed. Supplementary Figure S7. The expression difference of 8 genes. (A) Expression difference of 8 genes in cancer and adjacent cancer. (B) Expression difference of 8 genes in three molecular subtypes. Supplementary Table S1. Sample information of each data set. Supplementary Table S2. The clinical information of TCGA-STAD dataset and GSE cohort. Suppl [file 8933167.f1.zip › 8933167.f1/Supplementary Table S3.pdf]

Supplementary Table S3. The information of training group and test group.

Clinical Features	GSE-STAD train	GSE-STAD test	P
**OS**			
0	339	93	0.296
1	320	72	
**GSE**			
GSE26942	68	25	
GSE66229	241	59	0.206
GSE84437	350	81	
**Gender**			
female	205	53	0.8751
male	454	112	
